# Effects of Stresscopin on Rat Hypothalamic Paraventricular Nucleus Neurons *In Vitro*

**DOI:** 10.1371/journal.pone.0053863

**Published:** 2013-01-18

**Authors:** Chun-Ping Chu, Wen-Zhe Jin, Yan-Hua Bing, Qing-Hua Jin, Hiroshi Kannan, De-Lai Qiu

**Affiliations:** 1 Cellular Function Research Center, Yanbian University, Yanji City, Jilin Province, People's Republic of China; 2 Department of Physiology and Pathophysiology, College of Medicine, Yanbian University, Yanji City, Jilin Province, People's Republic of China; 3 Department of Pain, Affiliated Hospital of Yanbian University, Yanji City, Jilin Province, People's Republic of China; 4 Departments of Nutrition, Faculty of Home Economics, Kyushu Women's University, Kitakyushu, Japan; CNRS - Université Aix Marseille, France

## Abstract

The effects of stresscopin (SCP) on rat paraventricular nucleus (PVN) neurons were examined using whole-cell patch-clamp recordings and single-cell reverse-transcription multiplex polymerase chain reaction (SC-RT-mPCR) techniques. Under current-clamp conditions, bath application of SCP (100 nM) induced inhibition in 35.2% (37/105) of putative magnocellular neurons and 24.7% (20/81) of putative parvocellular neurons, and excitation in 5.7% (6/105) of putative magnocellular neurons and 18.5% (15/81) of putative parvocellular neurons. SCP-induced inhibition persisted in the presence of a mixture of TTX, a voltage-gated Na+ channel blocker, CNQX, an AMPA/kainate receptor antagonist and bicuculline, a GABA_A_ receptor antagonist, whereas SCP-induced excitation of PVN neurons was reversed by the mixture. The SCP-induced inhibition of PVN neurons was abolished by bath application of antisauvagine-30, a selective CRF receptor 2 (CRF-R2) antagonist. Under voltage-clamp conditions, SCP evoked outward currents at the holding potential (−60 mV), which reversed near the potassium equilibrium potential. The SCP-evoked membrane currents were completely blocked by bath application of tertiapin-Q, a selective blocker of G protein-activated inwardly rectifying potassium (GIRK) channels. SC-RT-mPCR analysis indicated that all the SCP-sensitive PVN neurons (57 SCP-inhibited neurons, 21 SCP-excited neurons) expressed CRF-R1 and CRF-R2 mRNAs. Among SCP-hyperpolarized PVN neurons, oxytocin (OT) mRNA was detected in 91.8% of putative magnocellular neurons and 45.0% of putative parvocellular neurons. OT mRNA was also detected in 26.6% of SCP-depolarized parvocellular neurons, but not in SCP-depolarized magnocellular neurons. These results indicate that SCP inhibits a subpopulation of PVN neurons, especially OTergic magnocellular neurons, by enhancing the activity of GIRK channels *via* CRF-R2.

## Introduction

Corticotropin-releasing factor (CRF), a 41-amino acid peptide, is synthesized and secreted in many regions of the central nervous system, and plays a key role in the coordination of endocrine and behavioral responses to stress. Human urocortin III, also known as stresscopin (SCP), is a 38-amino acid peptide of the CRF family [1], [2]. Two G-protein-coupled receptors, termed CRF receptor 1 (CRF-R1) and CRF receptor 2 (CRF-R2), have been identified as CRF receptors [3]. SCP is presumed to be a specific ligand for CRF-R2, and binds and activates the receptor [1], [3], [4]. *In vitro* binding studies have shown that SCP binds CRF-R2 with high affinity but has minimal affinity for CRF-R1, and it stimulates cAMP production in cells expressing CRF-R2, but not in cells expressing CRF-R1 [1], [2]. In contrast, CRF binds with high affinity to CRF-R1 [5]. CRF-R1 is highly expressed in the anterior pituitary, olfactory bulb, neocortex, hippocampus, amygdala and cerebellum [6]. CRF-R2 (α) is expressed mainly in the hypothalamic ventromedial nucleus and paraventricular nucleus (PVN), medial amygdaloid nucleus and lateral septic nucleus of the brain [7], and both CRF-R1 mRNA and CRF-R2 mRNA are expressed in putative parvocellular neurons in the PVN of rats [8].

The distribution of SCP-containing neurons and fibers in the rat brain has been investigated by *in situ* hybridization and immunohistochemistry [1], [2], [4]. SCP mRNA is expressed in major regions of the brain, including the rostral perifornical area of the hypothalamus, the lateral septum and the medial amygdaloid nucleus [1], [2]. In the hypothalamus, SCP-positive neurons are observed in the Median Preoptic Nucleus of rats [4], which project heavily to neurosecretory neurons of the PVN and the supraoptic nucleus [9], [10], suggesting that SCP may regulate the function of PVN neurosecretory neurons.

Central administration of SCP stimulates the hypothalamic-pituitary-adrenal axis, elevating levels of plasma adrenocorticotropic hormone. It also suppresses feeding [11]–[14], elevates blood glucose levels [11], [15], and elicits transient increases in mean arterial blood pressure and heart rate [16], [17]. Microinjection of SCP into the PVN induces significant increases in systemic blood pressure, heart rate and renal sympathetic nerve activity *via* CRF-R2 [18]. Although numerous studies indicate that central SCP modulates neurosecretory and cardiovascular function, the underlying mechanisms are currently unclear.

The PVN consists of magnocellular neurons, neurosecretory parvocellular neurons and non-neurosecretory preautonomic parvocellular neurons, which play a critical role in the regulation of stress responses and neurosecretory and autonomic functions [9], [19]. We investigated the effects of SCP on PVN neurons using whole-cell patch-clamp recordings, as well as pharmacological and SC-RT-mPCR techniques *in vitro* in rats. We found that SCP induced both inhibition and excitation in PVN neurons. SCP-induced inhibition persisted in the presence of a mixture of TTX, CNQX and bicuculline, whereas SCP-induced excitation of PVN neurons was reversed by bath application of the mixture. The SCP-induced inhibition was blocked by a selective CRF-R2 antagonist. The SCP-induced membrane currents reversed near the potassium equilibrium potential, and were blocked by a selective blocker of GIRK channels. SC-RT-mPCR analysis indicated that all the SCP-sensitive PVN neurons expressed CRF-R1 and CRF-R2 mRNA. Oxytocin (OT) mRNA was detected in 91.8% of putative magnocellular neurons, 45.0% of putative parvocellular neurons and 26.6% of SCP-depolarized parvocellular neurons.

## Results

### Effects of SCP on membrane potential

**Figure 1:**
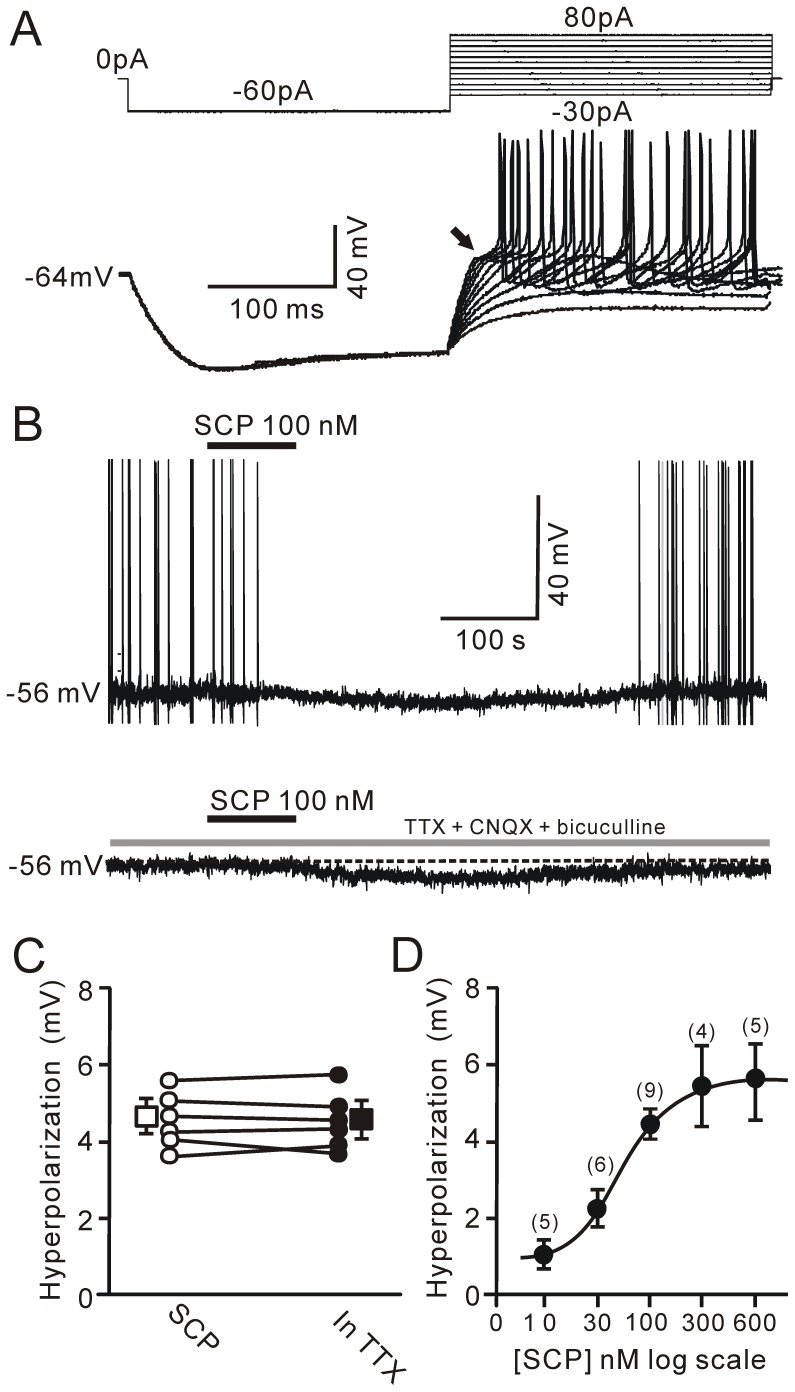
SCP directly inhibited the activity of a subpopulation of PVN neurons. A, Under current-clamp recording mode, a putative PVN magnocellular neuroendocrine neuron displayed a large inward rectification and strong transient outward rectification (black arrow) in response to a series of depolarizing current pulses delivered at a hyperpolarized membrane potential. B, Upper, representative response trace showing that SCP (100 nM) induced hyperpolarization of membrane potential and cessation of spike firing in a PVN neuron. Lower, SCP-induced hyperpolarization persisted in the presence of a mixture of 0.5 µM TTX, 10 µM CNQX and 10 µM bicuculline. C, Mean values (± SEM) and individual data showing that SCP-induced hyperpolarization was unaffected by the mixture of TTX + CNQX + bicuculline. D, Concentration-response curve showing that SCP induced a dose-dependent hyperpolarization. The number of neurons tested for each concentration is indicated near the bars.

**Figure 2:**
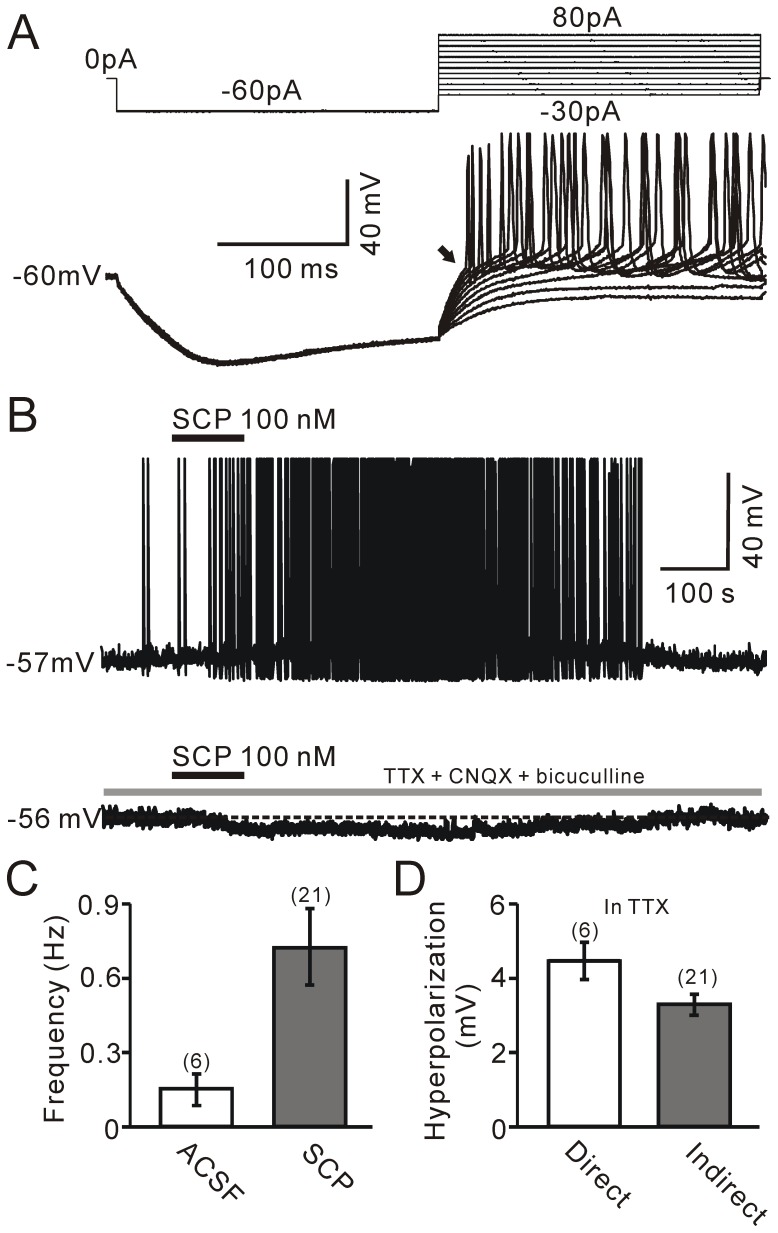
SCP excited indirectly a subpopulation of PVN parvocellular neurons. Under current-clamp recording mode, a putative PVN parvocellular neuroendocrine neuron displayed a lack of transient outward rectification (black arrow) in response to a series of depolarizing current pulses delivered at a hyperpolarized membrane potential. B, Upper, representative responses trace showing that SCP (100 nM) induced membrane depolarization and an increase in spike firing in the neuron. Lower, SCP induced a hyperpolarization of membrane potential when the mixture of 0.5 µM TTX, 10 µM CNQX and 10 µM bicuculline was present. C, Pooled data showing the effects of SCP (100 nM) on the spike firing rate of PVN neurons. D, Summary of data showing that SCP hyperpolarized membrane potential by directly inhibiting (Direct) and indirectly inhibiting (Indirect) a PVN neuron when a mixture of TTX, CNQX and bicuculline was present.

A total of 186 PVN neurons (56 rats) were recorded using the whole-cell patch-clamp recording technique. In addition, these cells were screened for GAPDH, CRF-R1, CRF-R2 and OT mRNA using the single-cell RT-mPCR method. Under current-clamp recording conditions, these PVN neurons were classified as either putative magnocellular neurons (105/186; Fig. 1A) or putative parvocellular neurons (81/186; Fig. 2A) according to previously established criteria [20], [21]. The effects of bath application of SCP for 90 s on both membrane potential and spike firing frequency of PVN neurons were examined after a 100-s stable baseline recording period. The application of SCP in concentrations ranging from 10 nM to 600 nM resulted in hyperpolarization of membrane potential and a decrease in spike firing rate in 35.2% (37/105) of putative magnocellular neurons and 24.7% (20/81) of putative parvocellular neurons, but without changing the characteristics of the action potentials (not shown). The hyperpolarization appeared at approximately 60 s and peaked at approximately 200 s after SCP application, with a mean peak value of 4.38±0.42 mV (Fig. 1B, C). The minimum dose of SCP required to elicit a significant effect on membrane potential was 10 nM, and the maximum dose was approximately 600 nM. To determine whether the SCP-induced hyperpolarization was due to a direct effect of SCP on PVN neurons, the amplitude of the SCP-induced hyperpolarization of membrane potential was determined in the absence or presence of a mixture of TTX (0.5 µM), CNQX (10 µM) and bicuculline (10 µM). In six SCP-inhibited PVN neurons, SCP induced a reversible hyperpolarization of membrane potential (Fig. 1B; upper). Following bath application of the mixture of TTX, CNQX and bicuculline for 10 min, reapplication of SCP induced a hyperpolarization of 4.41±0.48 mV, which was similar to SCP-induced hyperpolarization under control conditions (4.71±0.44 mV; *P* = 0.65; n = 6; Fig. 1B, C). The SCP-induced hyperpolarization was dose-dependent; the half maximal inhibitory concentration (IC_50_) was approximately 54 nM (Fig. 1D). These data indicate that bath application of SCP directly hyperpolarizes a subpopulation of PVN neurons. However, SCP (100 nM) induced depolarization of membrane potential (3.16±0.15 mV; n = 21) and an increase in spike firing rate in 5.7% (6/105) of putative magnocellular neurons and 18.5% (15/81) of putative parvocellular neurons (Fig. 2A, B). The frequency of spike firing increased from 0.19±0.08 Hz to 0.81±0.17 Hz (n = 21; *P* = 0.002; Fig. 2C). Intriguingly, bath application of a mixture of TTX, CNQX and bicuculline abolished the SCP-elicited depolarization of membrane potential and revealed the SCP-induced hyperpolarization of membrane potential in these neurons. In the presence of TTX, CNQX and bicuculline, SCP induced a hyperpolarization of membrane potential, with a mean value of 3.31±0.22 mV (n = 21), which was weaker than the evoked hyperpolarization of membrane potential in SCP-inhibited neurons (4.41±0.48 mV; n = 6; *P* = 0.04; Fig. 2D), suggesting that SCP-induced presynaptic effects overwhelmed the direct effect of SCP on the postsynaptic membrane. These results indicate that the SCP-elicited excitation of a subpopulation of PVN neurons (due to bath application of the peptide) was likely achieved indirectly through a circuitry effect. Indeed, SCP hyperpolarized SCP-excited neurons directly through the postsynaptic membrane.

### SCP-induced inhibition of PVN neurons is mediated by CRF-R2

**Figure 3:**
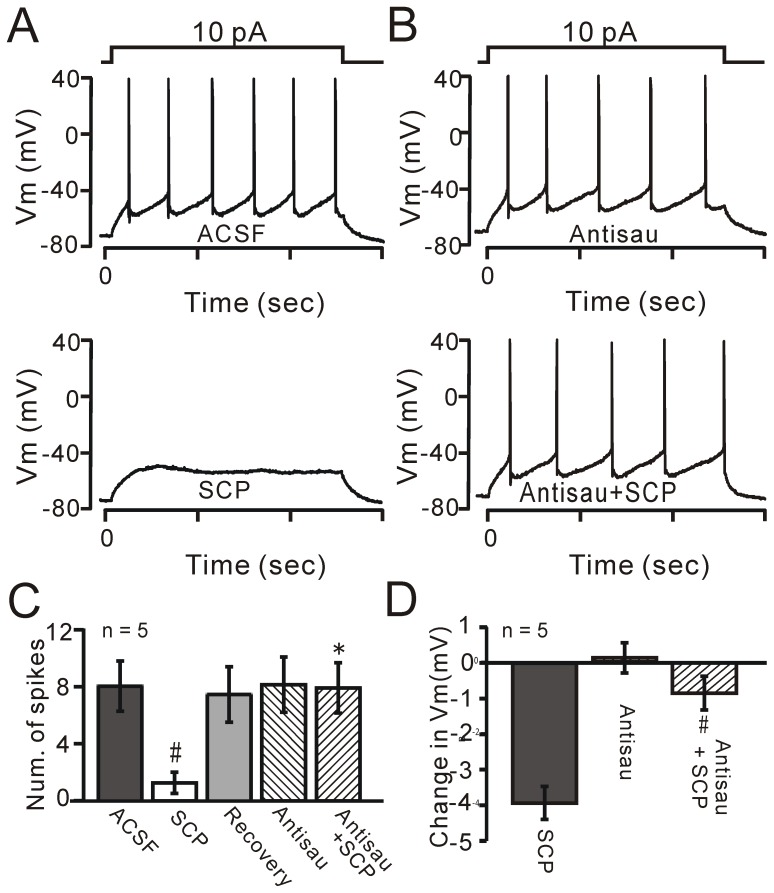
SCP-induced inhibition of PVN neurons is prevented by a selective CRF-R2 antagonist. A, Under current-clamp, spike firing evoked in a PVN neuron by injection of a small depolarizing current pulse (duration: 1 s; amplitude: 10 pA) in ACSF, SCP (100 nM) and Recovery (Re; grey). B, Antisauvagine-30, a selective CRF-R2 antagonist (30 nM), did not affect the evoked spike firing of the neuron (upper), but abolished the SCP-induced decrease in spike firing (lower). C, Pooled data (n = 5) showing the mean values of spike firing evoked by the depolarizing current pulse in ACSF, SCP (100 nM), antisauvagine-30 (30 nM) and antisauvagine-30 + SCP. ^#^ *P* = 0.0007 vs ACSF; ^*^ *P* = 0.0009 vs SCP. D, Summary of data showing the change in membrane potential (Vm) in neurons treated with SCP, antisauvagine-30, a mixture of antisauvagine-30 and SCP. Note that antisauvagine-30 prevented the SCP-induced hyperpolarization of membrane potential. ^#^ *P* = 0.02 vs SCP.

CRF receptors are classified as CRF-R1 or CRF-R2 [3]. CRF has high affinity for CRF-R1, whereas SCP is presumed to be a specific ligand for CRF-R2 [1], [3], [4]. Our results show that bath application of SCP directly hyperpolarizes a subpopulation of PVN neurons, and this effect may be mediated by CRF receptors, especially CRF-R2. To examine the pharmacological profile of SCP-induced inhibition mediated by CRF receptors, a CRF receptor nonselective antagonist, α-helical CRF-(9–14), or a selective and competitive CRF-R2 antagonist, antisauvagine-30, was applied to the SCP-inhibited neurons under current-clamp conditions [22], [23]. Bath application of α-helical CRF prevented the SCP-induced hyperpolarization of membrane potential in SCP-inhibited neurons (SCP: −4.11±0.42 mV; SCP + α-helical CRF: 0.61±0.39 mV; *P* = 0.004; n = 8; not shown). Since SCP is presumed to be a specific ligand for CRF-R2 [1], [3], [4], we hypothesized that SCP-elicited inhibition of PVN neurons is mediated by CRF-R2. To test this, we further examined the effects of SCP on neuronal excitability in the presence or absence of antisauvagine-30 (Fig. 3). SCP (100 nM) reversibly decreased the number of spikes elicited by a 1-s depolarizing current pulse under control conditions (Fig. 3A). The mean frequency of evoked spike firing decreased from 7.56±1.65 Hz to 1.19±0.70 Hz (*P* = 0.0007; n = 5; Fig. 3C). Bath application of antisauvagine-30 (30 nM) did not alter the number of spikes (7.65±1.82 Hz, *P* = 0.74 vs ACSF; n = 5), but abolished the SCP-induced reduction in the number of spikes (Fig. 3B); the mean frequency of evoked spikes was 7.44±1.66 Hz in the presence of the mixture of antisauvagine-30 and SCP (*P* = 0.0009 vs SCP; n = 5; Fig. 3C). Application of antisauvagine-30 did not alter the membrane potential (0.11±0.42 mV; n = 5; Fig. 3D), but prevented the SCP-induced hyperpolarization of membrane potential in SCP-inhibited neurons (SCP: 3.93±0.45 mV, n = 12; SCP + antisauvagine-30: 0.85±0.47 mV, n = 5; *P* = 0.02; Fig. 3D). These data indicate that the SCP-induced direct hyperpolarization in a subpopulation of PVN neurons is mediated by CRF-R2.

### SCP enhances GIRK channel activity under voltage-clamp

**Figure 4:**
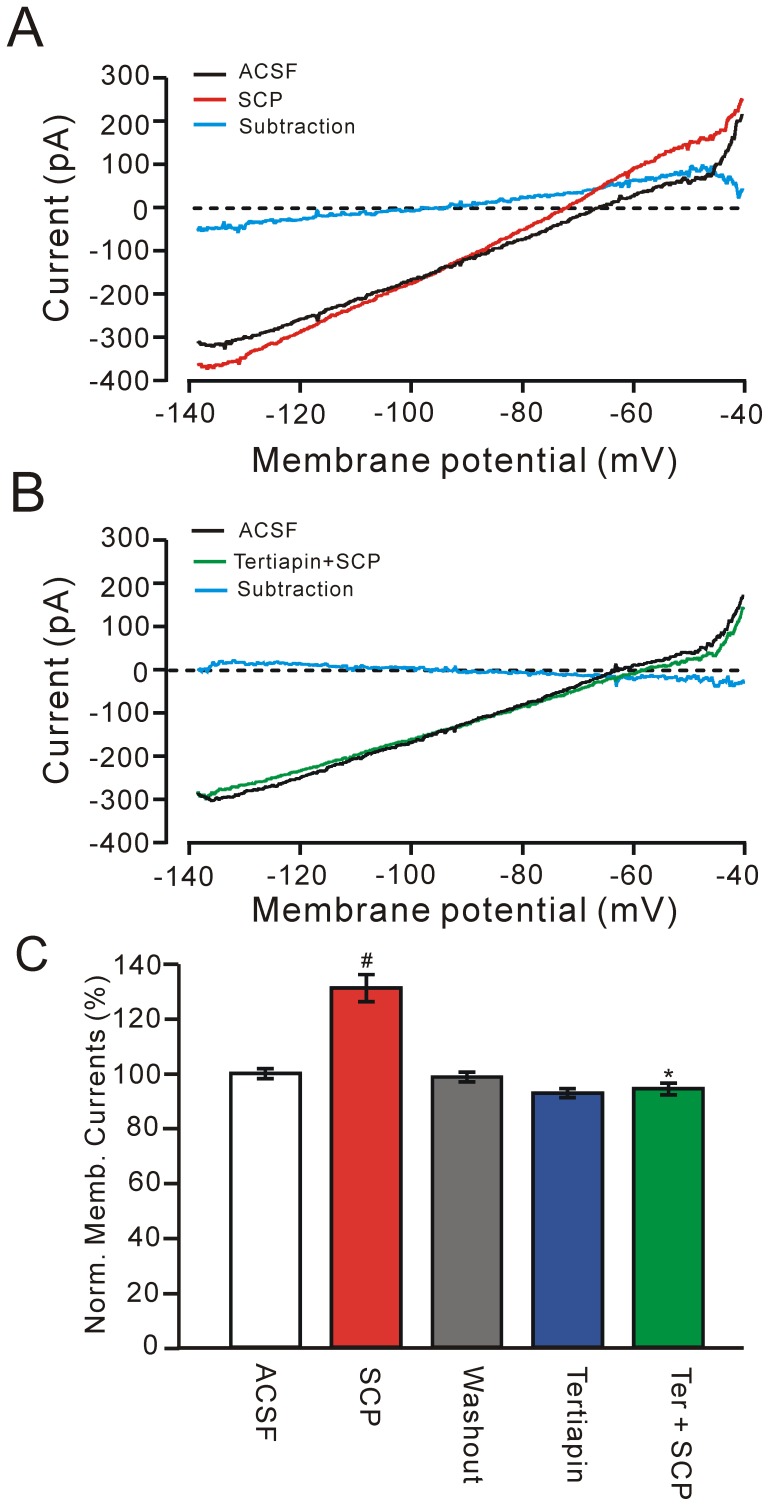
SCP enhances GIRK currents in PVN neurons. A, Steady-state (−140 to −40 mV; 20 mV/s; V_hold_ = −60 mV) whole-cell current-voltage relationships showing the effects of SCP (300 nM) on a PVN neuron. The subtracted trace (blue) shows the SCP-induced current. Note that SCP evokes outward currents at the holding potential (−60 mV) and is associated with an increase in membrane conductance (subtraction). The reversal potential of the evoked current is −98.5 mV. B, Whole-cell current-voltage relationships showing the effects of tertiapin-Q (200 nM), a selective GIRK channel blocker, on the SCP (300 nM)-induced current in the neuron shown in A. The subtracted trace (blue) shows the membrane currents evoked in the presence of the mixture of tertiapin-Q and SCP. Note that tertiapin-Q blocked the SCP-induced increase in membrane currents. C, Pooled data (n = 6) showing the normalized change of membrane currents at holding potential (-60 mV) when SCP, tertiapin-Q, and tertiapin-Q + SCP (300 nM) were present. ^#^ *P* = 0.036 vs ACSF; ^*^ *P* = 0.007 vs SCP.

Under voltage-clamp, the effects of SCP (300 nM) on SCP-sensitive PVN neurons were examined using steady-state (−140 to 40 mV, 20 mV/s; V_hold_ = −60 mV) whole-cell current-voltage (I–V) relationships. The I–V relationships revealed that 300 nM SCP evoked an outward current at the holding potential (−60 mV) that was associated with an increase in membrane conductance (Fig. 4A). The reversal potential of the evoked currents was −95.6±2.31 mV (n = 6; Fig. 4A, subtraction), which was near the potassium equilibrium potential calculated using the Nernst equation (E_k_ = −98.7 mV). When neurons were held at −60 mV, SCP induced an increase in outward current; the normalized membrane current was 130.5±3.7% of baseline (*P* = 0.023; n = 12). These results suggest that bath application of SCP induces an increase in membrane current through potassium channels.

Since CRF receptors are G protein-coupled receptors and SCP induces hyperpolarization by enhancing the activity of potassium channels, it is possible that SCP could hyperpolarize PVN neurons by activating G protein-activated inwardly rectifying potassium (GIRK) channels. To test this hypothesis, we applied a non-specific blocker of GIRKs, BaCl_2_, or a specific blocker, tertiapin-Q [24]. When neurons were held at −60 mV, SCP (300 nM) induced an increase in outward current, elevating it to 129.6±4.1% of baseline (*P* = 0.036; n = 6). Bath application of Ba^2+^ (0.2 mM) for 5 min completely blocked the SCP-induced increase in outward current (data not shown). Since Ba^2+^ is a relatively specific blocker of inwardly rectifying K^+^ channels (IRKs) and GIRKs at a concentration of 0.2 mM [24], we employed a selective blocker of GIRK channels, tertiapin-Q (200 nM). In neurons, tertiapin-Q blocks GIRK channels, with little effect on IRK channels [25]. Under voltage-clamp, the I-V relationships revealed that tertiapin-Q blocked the SCP-evoked membrane current (Fig. 4B, subtraction). When the neurons were held at −60 mV, bath application of tertiapin-Q induced a decrease in membrane current by 92.9±1.6% of baseline (*P* = 0.042; n = 6) and completely blocked the SCP-induced increase in the outward current (SCP: 131.3±5.0% of baseline; tertiapin-Q + SCP: 94.4±2.1% of baseline; n = 6; *P* = 0.007; Fig. 4C). These results indicate that extracellular blockade of GIRK channels prevents the SCP-induced increase in membrane current, suggesting that SCP-induced hyperpolarization of membrane potential was mediated by the activation of GIRK channels. In addition, application of tertiapin-Q blocked the activity of GIRK channels, resulting in a significant decrease in membrane current. This indicates that GIRK channels are constitutively activated at a holding potential of −60 mV [26], [27].

**Figure 5:**
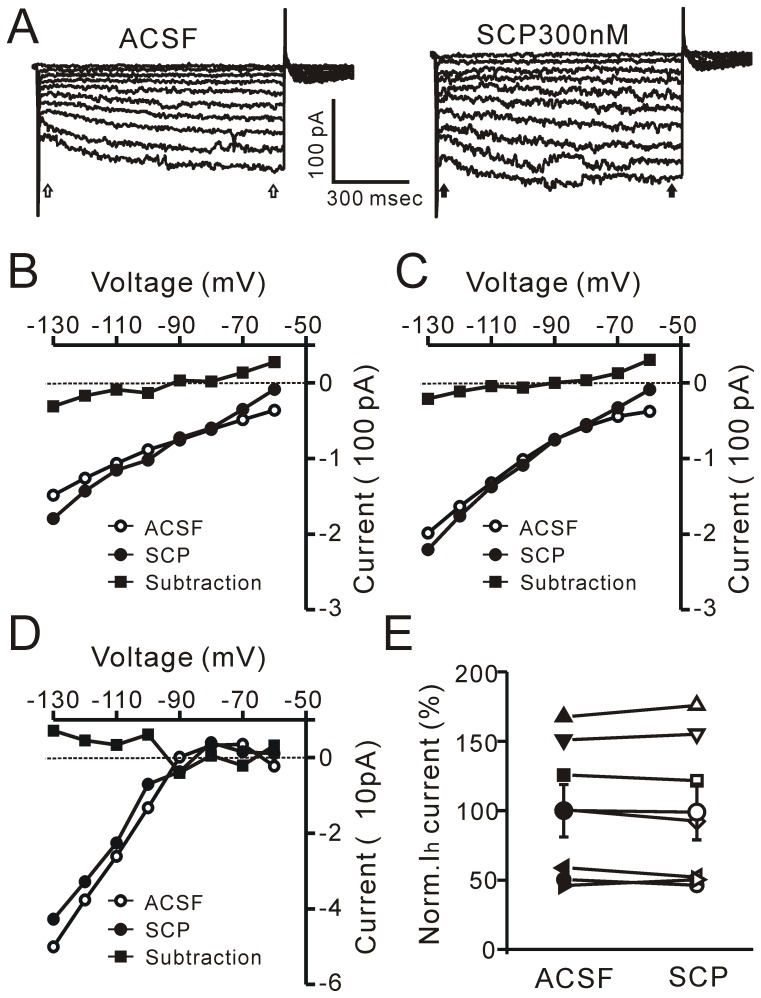
Effects of SCP on I_H_ currents in PVN neurons. A, Current traces elicited by a series of 1-s hyperpolarizing voltage steps (10 mV decrements; holding potential at −60 mV) in ACSF (left) and during the application of 300 nM SCP (right). B, Plots of instantaneous current (I_Ins_) in ACSF (○), during the application of SCP (SCP; •) and subtraction of SCP from control (□) against the membrane potential (↑ shown in A). C, Plots of the steady-state current (I_SS_) in ACSF (○), during the application of SCP (•) and subtraction of SCP from control (□) against the membrane potential (↑ shown in A). D, Plots of the I_H_ (I_SS_ – I_Ins_) in the control (○), during the application of SCP (•) and subtraction of SCP from control (□) against the membrane potential. E, Mean values (± SEM; n = 7) and individual data showing the I_H_ in ACSF and SCP.

CRF augments I_H_ *via* CRF-R1 [21], [28], [29]. Therefore we examined the effects of SCP on I_H_ under voltage clamp conditions. In the presence of TTX (0.5 µM), bath application of SCP (300 nM) produced an outward current (30.8±4.6 pA; n = 7) at a holding potential of −60 mV. When a series of 1-s hyperpolarizing voltage steps from −60 mV to −130 mV were applied, SCP induced an increase in instantaneous current (I_Ins_) and steady-state current (I_SS_), which were reversed at 92.4±4.36 mV (I_Ins_; n = 7; Fig. 5A, B) and 94.3±3.11 mV (I_SS_; n = 7; Fig. 5A, C), respectively. The reversal potentials of I_Ins_ and I_SS_ were near the potassium equilibrium potential calculated by the Nernst equation (E_k_ = −98.7 mV). However, SCP did not change the amplitude of the I_H_ (I_SS_ – I_Ins_) current (Fig. 5D); the normalized values of I_H_ evoked by the 1-s hyperpolarizing voltage (−130 mV) were 100.0±18.8% in artificial cerebrospinal fluid (ACSF) and 99.0±20.1% in the presence of SCP (Fig. 5E). These results indicate that SCP augments the activity of GIRK channels without affecting the I_H_ current.

### SCP-inhibited PVN neurons express OT mRNA

**Figure 6:**
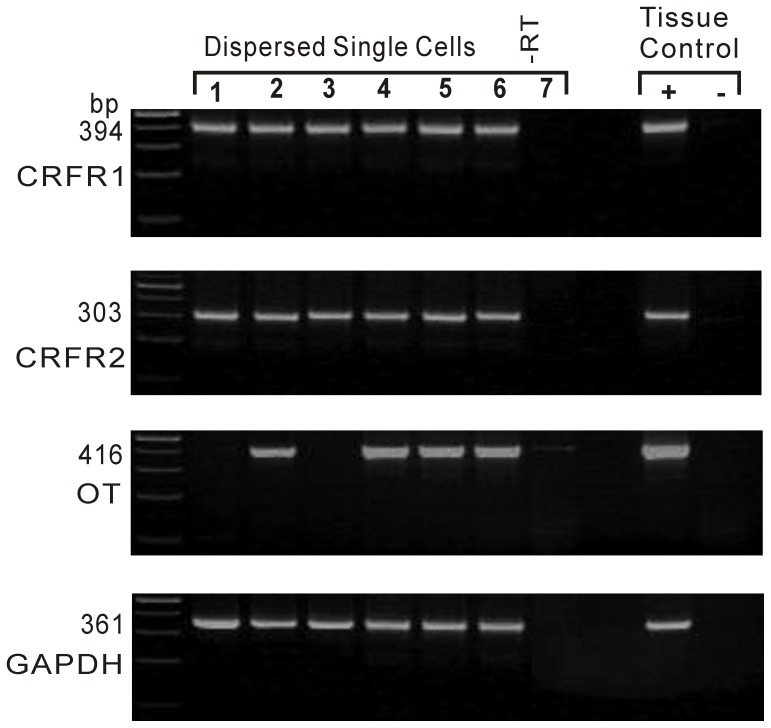
SCP-inhibited neurons express CRF-R1, CRF-R2 and OT mRNA. The positive control (Tissue Control +) showed that mRNAs for CRF-R1, CRF-R2, OT and GAPDH were detected in rat hypothalamic tissue total RNA. GAPDH transcripts were analyzed in the same cells as an internal control for the RT reaction. The expected sizes of the PCR products are indicated. Cells 1 and 2 are putative parvocellular neurons, while cells 3–6 are putative magnocellular neurons. Four of 7 cells expressed OT mRNA. In addition, a single cell (-RT) and rat hypothalamic tissue total RNA (Tissue Control -) were processed without RT, but no PCR products were obtained.

After completion of electrophysiological recordings, the cytoplasm was aspirated into the patch pipette by the application of a gentle negative pressure, and first-strand cDNA was synthesized. All the SCP-sensitive neurons were screened for GAPDH (positive control), CRF-R1, CRF-R2 and OT mRNA using the SC-RT-mPCR technique. Screening of rat hypothalamic total RNA (positive control) resulted in detection of all the specific mRNAs, each corresponding to the size predicted by its mRNA sequence. The SC-RT-mPCR analysis indicated that all the SCP-sensitive neurons expressed CRF-R1 and CRF-R2 mRNAs (Fig. 6). Among the PVN neurons directly hyperpolarized by SCP, OT mRNA was detected in 91.8% (34/37) of putative magnocellular neurons and 45.0% (9/20) of putative parvocellular neurons (Fig. 6). In comparison, OT mRNA was detected in only 26.6% (4/15) of SCP-depolarized parvocellular neurons, and not at all in SCP-depolarized magnocellular neurons.

## Discussion

SCP, also known as human urocortin III, is considered a specific ligand for CRF-R2. *In vitro* studies have shown that SCP binds CRF-R2 with high affinity [1], [2]. Although the actions of SCP have been investigated for some time, this is the first study, to our knowledge, examining the effects of the peptide on PVN neuronal membrane potential and currents. In this study, we found that bath application of SCP induced inhibition in 35.2% of putative magnocellular neurons and 24.7% of putative parvocellular neurons, and excitation in 5.7% of putative magnocellular neurons and 18.5% of putative parvocellular neurons. SCP-induced inhibition, but not excitation, persisted in the presence of a mixture of TTX, CNQX and bicuculline. The SCP-mediated inhibition was blocked by a selective CRF-R2 antagonist. Under voltage-clamp, SCP evoked outward currents that reversed near the potassium equilibrium potential and that could be blocked by the selective GIRK channel blocker. SC-RT-mPCR analysis indicated that all SCP-sensitive PVN neurons expressed CRF-R1 and CRF-R2 mRNA. Approximately 70% of SCP-hyperpolarized neurons expressed OT mRNA. These results indicate that SCP inhibits a subpopulation of PVN neurons, especially OTergic magnocellular neurons, by enhancing the activity of GIRK channels *via* CRF-R2.

Both mouse and human SCP are highly selective for CRF-R2 and exhibit low affinities for CRF-R1 [1], [3], [4]. In the brain, CRF-R2 (α) is expressed mainly in the hypothalamic PVN, medial amygdaloid nucleus and lateral septic nucleus of the brain [7]. We previously found that PVN neurons express CRF-R1 and CRF-R2 mRNAs, and that CRF depolarizes a subpopulation of PVN parvocellular neurons *via* CRF-R1 [8]. In this study, our SC-RT-mPCR results confirmed that SCP-sensitive neurons co-express CRF-R1 and CRF-R2 mRNAs, suggesting that CRF-R1 and CRF-R2 receptors are both present in SCP-inhibited PVN neurons. Furthermore, the SCP-elicited hyperpolarization of membrane potential in PVN neurons was blocked by the CRF-R1 and CRF-R2 non-selective antagonist, α-helical CRF [22], and by a selective CRF-R2 antagonist, antisauvagine-30 [23]. This indicates that the SCP-induced hyperpolarization was mediated by CRF-R2. Our results are consistent with *in vitro* binding studies showing that SCP binds CRF-R2 with high affinity, but has minimal affinity for CRF-R1 [1], [2]. This suggests that the direct inhibition of the subpopulation of PVN neurons by SCP is mainly mediated through CRF-R2.

PVN neurons receive glutamatergic and GABAergic afferents from a number of forebrain structures, including the Median Preoptic Nucleus, the subfornical organ, the Organum Vasculosum of the Lamina Terminal, as well as brainstem nuclei [19] and other intrahypothalamic nuclei [30]. In the present study, SCP induced excitation in 5.7% of putative magnocellular neurons and 18.5% of putative parvocellular neurons. Interestingly, the SCP-induced depolarization was reversed by the mixture of TTX, CNQX and bicuculline, suggesting that SCP might excite a subpopulation of PVN neurons through an indirect pathway; for example, through activation of glutamatergic interneurons or inhibition of GABAergic interneurons. Activation of glutamatergic interneurons can increase glutamate release onto PVN neurons, resulting in membrane depolarization and an increase in spike firing rate. However, the vast majority of local synaptic inputs onto PVN neurons are GABAergic [31]. Despite additional excitatory glutamatergic inputs [32], PVN neurons receive tonic GABAergic inhibition, which maintain low rates of spontaneous firing [33], [34]. Inhibition of GABAergic interneurons can reduce tonic and phasic inhibition of PVN neurons, resulting in membrane depolarization and an increase in firing rate.

### SCP promotes the activation of GIRK channels

The GIRK channels, which are members of the IRK channel family, are directly activated by G proteins and are considered to play a critical role in the inhibitory regulation of neural activity. Electrophysiological studies have shown that a variety of G protein-coupled receptors, including M_2_-muscarinic, D_2_-dopamine, GABA_B_, opioid and somatostatin receptors in the brain, interact with GIRK channels to induce inhibitory postsynaptic potentials [35]. In the hypothalamic PVN, pharmacological activation of GABA_B_ receptors produces inhibition of magnocellular neurons *via* activation of GIRK channel conductance [36]. Bath application of the α_2_-adrenoceptor agonist dexmedetomidine inhibits PVN magnocellular neurons through activation of GIRKs and suppression of hyperpolarization-activated currents [37]. In this study, I-V relationships revealed that SCP evoked outward currents when membrane potential was held at −60 mV, and the reversal potential of the evoked currents was near the potassium equilibrium potential. These results indicate that bath application of SCP induces an increase in outward currents through potassium channels. Furthermore, the SCP-evoked increase in outward currents was completely blocked by bath application of Ba^2+^, a non-selective blocker of IRK and GIRK channels [24], [37]. Since both CRF-R1 and CRF-R2 are G protein-coupled receptors, it is most likely that SCP hyperpolarizes PVN neurons by enhancing GIRK channel activity. Collectively, our results show that blockade of GIRK channels prevents the SCP-induced increase in membrane currents, suggesting that SCP activates GIRK channels *via* CRF-R2, resulting in the hyperpolarization of membrane potential. Further studies are required to clarify the signaling pathways and molecular mechanisms underlying the SCP-induced activation of GIRK channels.

### Physiological significance

The PVN is a complex heterogeneous region consisting of magnocellular neurons and parvocellular neurons. It has the critical task of integrating endocrine and autonomic functions, including the stress response and autonomic control of cardiovascular activity [19]. PVN magnocellular neurons integrate incoming information and secret OT and vasopressin from their nerve terminals in the posterior pituitary, whereas PVN parvocellular neurons comprise neuroendocrine neurons and pre-autonomic neurons [9]. In the hypothalamus, SCP-positive neurons are observed in the Median Preoptic Nucleus of rats, suggesting that SCP secreted from this region may regulate the neurosecretory function of PVN neurons *via* CRF-R2. Our present results show that SCP mediates inhibition of OTergic neurons by enhancing GIRK channel activity *via* CRF-R2. OT plays an important role in mediating stress responses. Various stressors, such as force swimming stress, shaker stress and chronic homotypic stress, increase OT mRNA expression or OT secretion in the PVN [38]–[40]. Increased levels of OT might modulate the stress response by attenuating stress-induced activation of the hypothalamus-pituitary-adrenal axis [41], [42]. Central administration of OT has been shown to attenuate stress-induced corticosterone release and create an anxiolytic effect in rats [42], [43]. Intracerebroventricular injection of OT significantly attenuates the increase in CRF mRNA expression in the PVN induced by restraint stress [40]. In the present study, we found that SCP inhibits OTergic neuronal activity *via* CRF-R2, whereas CRF depolarizes OTergic neurons by enhancing I_H_ channel activity *via* CRF-R1 [21].

PVN parvocellular neurons comprise neuroendocrine neurons and pre-autonomic neurons, including CRF-secreting neurons and spinally projecting neurons, which play a critical role in the regulation of endocrine and autonomic functions [9], [19]. Approximately 25% of PVN parvocellular neurons are directly inhibited by SCP through enhancement of GIRK channel activity *via* CRF-R2, suggesting that SCP might attenuate stress and autonomic responses. In addition, the vast majority of local synaptic inputs to PVN neurons are GABAergic [31]. PVN neurons receive tonic GABAergic inhibition, which maintain their low rates of spontaneous spiking [33], [34]. Our results demonstrate that SCP indirectly depolarizes a subpopulation of PVN neurons, suggesting that SCP also directly inhibits GABAergic interneurons *via* CRF-R2, resulting in a decrease in tonic and phasic inhibition of PVN neurons. The decrease in tonic inhibition of spinally projecting sympathetic pre-autonomic neurons likely results in an increase in sympathetic outflow [44]. Consequently, central administration of SCP transiently increases mean arterial blood pressure, heart rate and epinephrine release [16]. Furthermore, microinjection of SCP into the PVN significantly increases systemic blood pressure, heart rate and renal sympathetic nerve activity *via* CRF-R2 [18].

## Materials and Methods

### Hypothalamic slice preparation

Hypothalamic slices were prepared from P12–14 male Wistar rats, as previously described [21], [45]. All experiments were approved by the Ethics Committee of Miyazaki University or the Animal Care and Use Committee of Jilin University, and were in accordance with international guidelines on the ethical use of animals in laboratory experiments. In brief, the brain was immediately placed into ice-cold oxygenated ACSF containing the following (in mM): 140 NaCl, 3 KCl, 1.3 MgSO_4_, 1.4 NaH_2_PO_4_, 5 2-[4-(2-hydroxyethyl)-1-piperazinyl]ethanesulfonic acid (HEPES), 11 D-glucose, 2.4 CaCl_2_ and 3.25 NaOH. The pH of the ACSF was 7.3, the osmolarity was 290–300 mOsm/L, and the solution was bubbled with 100% O_2_. Coronal hypothalamic slices (250 µm thick) were prepared using a vibrating brain slicer (DSK-2000; Dosaka, Kyoto, Japan). The slices were incubated for at least 1 hour in a chamber filled with equilibrated ACSF at room temperature (24–26°C) before electrophysiological recordings were started.

### Electrophysiology

Patch pipettes were made from thick-wall borosilicate glass (GD-1.5; Narishige) using a puller (PB-7; Narishige, Tokyo, Japan). They were filled with a solution consisting of (in mM) 130 potassium gluconate, 10 HEPES, 10 KCl, 5 EGTA, 1 CaCl_2_, 1 MgCl_2_, 2 Na_2_ATP and 0.5 Na_3_GTP. The pH was adjusted to 7.2 with KOH. Patch pipette resistances were 5–7 M*Ω* in the bath, with series resistances in the range of 10–20 M*Ω*. Membrane potentials and/or currents were monitored with an Axopatch 200B amplifier or an Axopatch −1D amplifier (Molecular Devices, Foster City, CA, USA), filtered at 5 kHz, and acquired through a Digidata 1200 series analog-to-digital interface on a personal computer using Clampex 7.0 or 8.1 software (Molecular Devices). Whole-cell recordings from PVN neurons were made from microscopically identified cells. Once stable recording conditions were obtained, PVN neurons were identified electrophysiologically as type I (magnocellular) or type II (parvocellular), according to previously established criteria by current-clamp in standard ACSF; type-I neurons displayed transient outward rectification, while type-II neurons did not [20], [21]. All the SCP-sensitive neurons were recorded from individual PVN slices.

Reagents included human SCP (Peptide Institute, Inc., Japan); anti-sauvagine-30 and tertiapin-Q (Tocris Bioscience, Bristol, UK); and α-helical CRF-(9–14), tetrodotoxin (TTX), 6-cyano-7-nitroquinoxaline-2,3-dione (CNQX), bicuculline and BaCl_2_ (Sigma-Aldrich, St. Louis, MO, USA). All drugs were dissolved in ACSF. In voltage-clamp, TTX (0.5 µM) was routinely included in external recording solutions to block voltage-gated Na^+^ channels.

### Cytoplasm harvesting and reverse transcription

Harvesting of cytoplasm and reverse transcription were carried out as previously described [21], [45]. After whole-cell recording, the cytoplasm was aspirated into the patch pipette by the application of a gentle negative pressure in the pipette while maintaining the tight seal. First strand cDNA was synthesized for 1 h at 42°C. The single-cell cDNA was kept at −70°C until PCR amplification.

### Multiplex and nested PCR

PCR amplification was performed with a thermal cycler (Gene Amp PCR system 9700; PerkinElmer, Norwalk, CT, USA) using a fraction (4 µl) of the single-cell cDNA as template. First multiplex-PCR was performed as a hot start in a final volume of 30 µl containing 4 µl cDNA, 100 pmol of each primer, 0.3 mM each dNTP, 3 µl 10× PCR buffer and 3.5 U HotStarTaq DNA Polymerase (Qiagen K.K., Tokyo, Japan) in a Gene Amp PCR system 9700 with the following cycling protocol: (1) 15 min at 95°C; (2) 35 cycles of 1 min at 94°C, 1.5 min at 57°C and 2 min at 72°C; (3) 10 min at 72°C; and (4) final hold at 4°C. The nested-PCR amplifications were carried out using 2.5 µl of the first PCR product with the following modification: 2.5 U HotStarTaq DNA Polymerase and 0.2 mM dNTPs. Thermocycling for the second round was as follows: (1) 15 min at 95°C; (2) 35 cycles of 45 s at 94°C, 1 min at 56°C and 1 min at 72°C; (3) 10 min at 72°C; and (4) final hold at 4°C.

The following nested primer sequences for GAPDH, CRFR-1, CRF-R2 and OT were used for SCP-inhibited neurons: GAPDH (accession No. NM_017008) external sense: 5′-GATGGTGAAGGTCGGTGTG (position 849), external antisense: 5′-GGGCTAAGCAGTTGGTGGT (position 1318); GAPDH internal sense: 5′-TACCAGGGCTGCCTTCTCT, internal antisense: 5′-CTCGTGGTTCACACCCATC (361 bp); CRFR-1 (accession No. NM_030999) external sense: 5′-GCCGCCTACAATTACTTCCA (position 1299), external antisense: 5′-GGACTGCTTGATGCTGTGAA (position 1958); CRFR-1 internal sense: 5′-GTGGATGTTCGTCTGCATTG, internal antisense: 5′-CACAAAGAAGCCCTGAAAGG (394 bp); CRF-R2 (accession No. NM_022714) external sense: 5′-TACTGCAACACGACCTTGGA (position 330), external antisense: 5′-ACCAGCACTGCTCATTCTCA (position 982); CRF-R2 internal sense: 5′-CCCTAGTGGAGAGACCATGC, internal antisense: 5′-AGGTGGTGATGAGGTTCCAG (303 bp); OT (accession No. NM_012996) external sense: 5′-ACACACCAGAAGAGGGCATC (position 1814), external antisense: 5′-GTCAGAGCCAGTAGGCCAAG (position 2580); OT internal sense: 5′-AGGGCCTTTGGTAGAGCAGT, internal antisense: 5′-GAGCTCAAAAGGGACACAGC (416 bp). To investigate the presence and size of the amplified fragments, 10-µl aliquots of PCR products were separated and visualized in an ethidium bromide-stained agarose gel (2%) following electrophoresis. All individual PCR products were verified several times by direct sequencing using BigDye Terminator v3.1 Cycle Sequencing Kit and an Applied Biosystems PRISM 310 Genetic Analyzer (ABI, Foster City, CA, USA). Sequence comparisons were performed using BLAST.

### RNA isolation and cDNA preparation for control reactions

Poly(A)^+^ RNA was prepared from fresh hypothalamus of 13-day-old Wistar rats using Micro-to-Midi Total RNA Purification System (Invitrogen). Reverse transcription was performed using 250 µg of the poly(A)^+^ RNA as described above. The positive controls were conducted in parallel with single-cell PCR amplification. The negative controls were performed in parallel with the single-cell experiments (excluding only the harvesting procedure) and resulted in no detectable bands (n = 10).

### Statistics

Electrophysiological data were analyzed using Clampfit 8.0 (Molecular Devices) and are expressed as mean ± SEM. The hyperpolarization-activated cation current (I_H_) was determined by subtracting I_Ins_ from I_ss_ at each hyperpolarizing voltage step using the following equation:
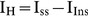
(1)


Differences between the mean values recorded under control and test conditions were evaluated with Student's paired t-test or analysis of variance using SPSS (Chicago, IL) software. Mean differences were considered to be significant at *P*<0.05.
